# *WebCSD*: the online portal to the Cambridge Structural Database

**DOI:** 10.1107/S0021889810000452

**Published:** 2010-02-12

**Authors:** Ian R. Thomas, Ian J. Bruno, Jason C. Cole, Clare F. Macrae, Elna Pidcock, Peter A. Wood

**Affiliations:** a Cambridge Crystallographic Data Centre, UK

**Keywords:** *WebCSD*, computer programs, database searching, Cambridge Structural Database, similarity searching, substructure, reduced cell

## Abstract

*WebCSD*, a new web-based application developed by the Cambridge Crystallographic Data Centre, offers fast searching of the Cambridge Structural Database using only a standard internet browser. Search facilities include two-dimensional substructure, molecular similarity, text/numeric and reduced cell searching. Text, chemical diagrams and three-dimensional structural information can all be studied in the results browser using the efficient entry summaries and embedded three-dimensional viewer.

## Abstract 2

The new web-based application *WebCSD* is introduced, which provides a range of facilities for searching the Cambridge Structural Database within a standard web browser. Search options within *WebCSD* include two-dimensional substructure, molecular similarity, text/numeric and reduced cell searching.

## 1. Introduction

*WebCSD* is a novel web-based application developed by the Cambridge Crystallographic Data Centre (CCDC). The software provides access to the information stored within the Cambridge Structural Database (CSD; Allen, 2002 ▶) using only a standard internet browser. *WebCSD* offers tools for searching, browsing and viewing crystal structures without the need to install any local software. *WebCSD* will allow simpler and more efficient dissemination of the CSD’s collection of small-molecule crystal structures, which is comprehensive for the published literature and contains many otherwise unpublished structures. It also provides access to the latest information through weekly updates to the public *WebCSD* servers. *WebCSD* provides an intuitive interface to fast, straightforward searches of the database, rather than attempting to replicate the extensive functionality of the (locally installed) CSD system software for structural analysis. This new application also contains some additional capabilities not accessible through the installed software, including similarity searching.

## 2. Overview

The software for searching crystal structure knowledge provided by the CCDC, such as *ConQuest* (Bruno *et al.*, 2002 ▶) and *Mercury* (Macrae *et al.*, 2008 ▶), has been focused on providing sophisticated and flexible tools for crystallographers, structural chemists and the drug design community. As the use of crystal structure information has broadened, more users are extra- or multi-disciplinary. This means that the demand for a more accessible and collaborative CSD user environment has increased. The Web provides the ideal medium for large companies and academic departments where communication and collaboration as well as software distribution can be challenging (Williams, 2008 ▶).

Areas where *WebCSD* is designed to be useful are in the medicinal chemistry and pharmaceutical arenas. Providing easy-to-use web-based tools for searching both in-house and CSD structures gives the chemist almost instant access to a wealth of valuable structural and conformational information (Taylor, 2002 ▶) without the need for locally installed software or lengthy start-up times sometimes associated with more complex tools. *WebCSD* is available *via* the CCDC’s public server and also as an intranet version that supports the use of in-house databases.

Another particularly important application of web software is in the area of chemical education. Knowledge of the three-dimensional nature of chemical compounds is fundamental to the education of every chemist (Bodner & Guay, 1997 ▶). Without this knowledge, concepts such as conformation, stereochemistry, chirality and the shapes of metal coordination environments cannot be properly understood. The CSD is therefore an essential resource for chemistry teachers. Not only does the CSD allow students to visualize and examine molecules in three dimensions, but it also provides an opportunity to work with real measured data, complete with experimental errors and statistical variations. Raw data mined from the CSD challenges students to think critically about the fundamental topics of bonding and molecular structure and also encourages them to consider the limitations and advantages of experimental structures. The ease of access offered *via WebCSD* should make it ideal for teachers and students to use the CSD in the classroom. In addition a 500-structure teaching subset of the database, containing a diverse set of molecules as well as a range of illustrative teaching exercises, is freely available online (Battle *et al.*, 2010 ▶).

## 3. Technical advances

### 3.1. Replacing the underlying database

Since the late 1980s, data within the CSD have been stored in the ASER format that was developed for use with the *QUEST* search program (Allen *et al.*, 1991 ▶), the precursor to *ConQuest*. The ASER format was designed to store bibliographic, connectivity and three-dimensional coordinate data together in a single record that was machine readable and efficient in terms of rapid access and low storage space. However, the structure of the ASER records has limited extensibility and it has become desirable to include additional data to address the needs of new areas of research.

*WebCSD* therefore uses the embedded relational database management system SQLite (http://www.sqlite.org). The SQLite system allows the addition of extra relational data tables, in which information can be sorted, that are linked to the CSD entries. These indexed data tables mean that searches can be sped up significantly by using a binary search algorithm which makes progressively better guesses to narrow down the search. The new system also allows easy and flexible storage of molecular fingerprints and database bit screens (Allen *et al.*, 1991 ▶) which are used in both substructure and similarity searching.

This paper describes *WebCSD* – the new web interface developed for searching and browsing the CSD – but the re-design of the underlying database system also allows access to the data *via* other web services. This will allow easy integration of the CSD with other online databases.

### 3.2. Multi-threading

*WebCSD*’s server-based software was designed from the outset to utilize multi-threading – this means that each individual search process and database query operates independently in a parallel computational process. The *WebCSD* server can therefore obtain maximum benefit from the computational resources available, which allows it to run faster on multi-core or multiple CPU systems. Another major advantage of this system, even using a single CPU, is that it allows the application to remain responsive to input whilst simultaneously executing multiple tasks. This means that users accessing *WebCSD* servers can run multiple, complex searches whilst concurrently browsing the results of an earlier completed search.

## 4. *WebCSD* search functionality

### 4.1. Server implementation

The search software that runs on the *WebCSD* servers is written in C++, using functionality provided by the CCDC’s C++ Toolkit (Bruno *et al.*, 2002 ▶). This same software is central to the CCDC’s *Mercury* (Macrae *et al.*, 2008 ▶), *Mogul* (Bruno *et al.*, 2004 ▶), *IsoStar* (Bruno *et al.*, 1997 ▶) and *enCIFer* (Allen *et al.*, 2004 ▶) applications.

### 4.2. Substructure searching

Substructure searching in *WebCSD*, and also in *ConQuest*, is achieved by decoding the search query into chemically meaningful information (*e.g.* contains Cl, or has a C=N bond), screening out structures that cannot possibly match (*i.e.* structures that do not contain the required component), and then comparing the connectivities (or molecular graphs) of the query and the remaining structures to determine matches.

Substructure searching (*i.e.* subgraph isomorphism) in the Toolkit was originally performed (Chisholm & Motherwell, 2004 ▶) with an in-house implementation of the Ullmann (1976 ▶) algorithm for depth-first searching. A re-implementation of the substructure searching code using a breadth-first backtracking approach has improved the performance when searching for particularly complex structures. This new implementation also stores less data at each stage of the search, giving a further improvement in performance. Additional optimizations were required for searches of large macrocyclic compounds, for example detecting whether the query contains a ring assembly larger than any that occur in the structures being searched: if so, there cannot be a match. Many new screens have also been introduced, which improve search speed noticeably by reducing the number of structures needing to be extracted from the database and searched.

The Toolkit’s substructure searching implementation was designed to be very flexible, making it easy to add new types of constraint. This has allowed *WebCSD* to offer some novel search options within the query sketcher, for example in dealing with cyclicity. In *WebCSD* it is possible to apply constraints with respect to the size of the smallest ring an atom or bond is involved in, such as the maximum, the minimum or a custom-defined range.

The *WebCSD* user interface currently allows two-dimensional searching (no intermolecular interactions or other three-dimensional constraints) and the software can identify matches very quickly as a result of the new algorithm. Three-dimensional searching will be added in a future version of the software.

### 4.3. Similarity searching

Alongside the substructure search tool is a complementary structure-based search option which determines the similarity of mol­ecular components in the CSD to a defined query molecule. There are two main aspects to the calculation of similarity between molecules in two dimensions: first the method by which the molecules are represented (commonly as ‘fingerprints’ or binary strings) and secondly the way in which the similarity between these representations is quantified (the similarity coefficient). The effectiveness of any similarity searching tool for a particular problem will be dependent on both of these aspects (Willett, 1987 ▶; Johnson & Maggiora, 1990 ▶).

**Figure 1:**
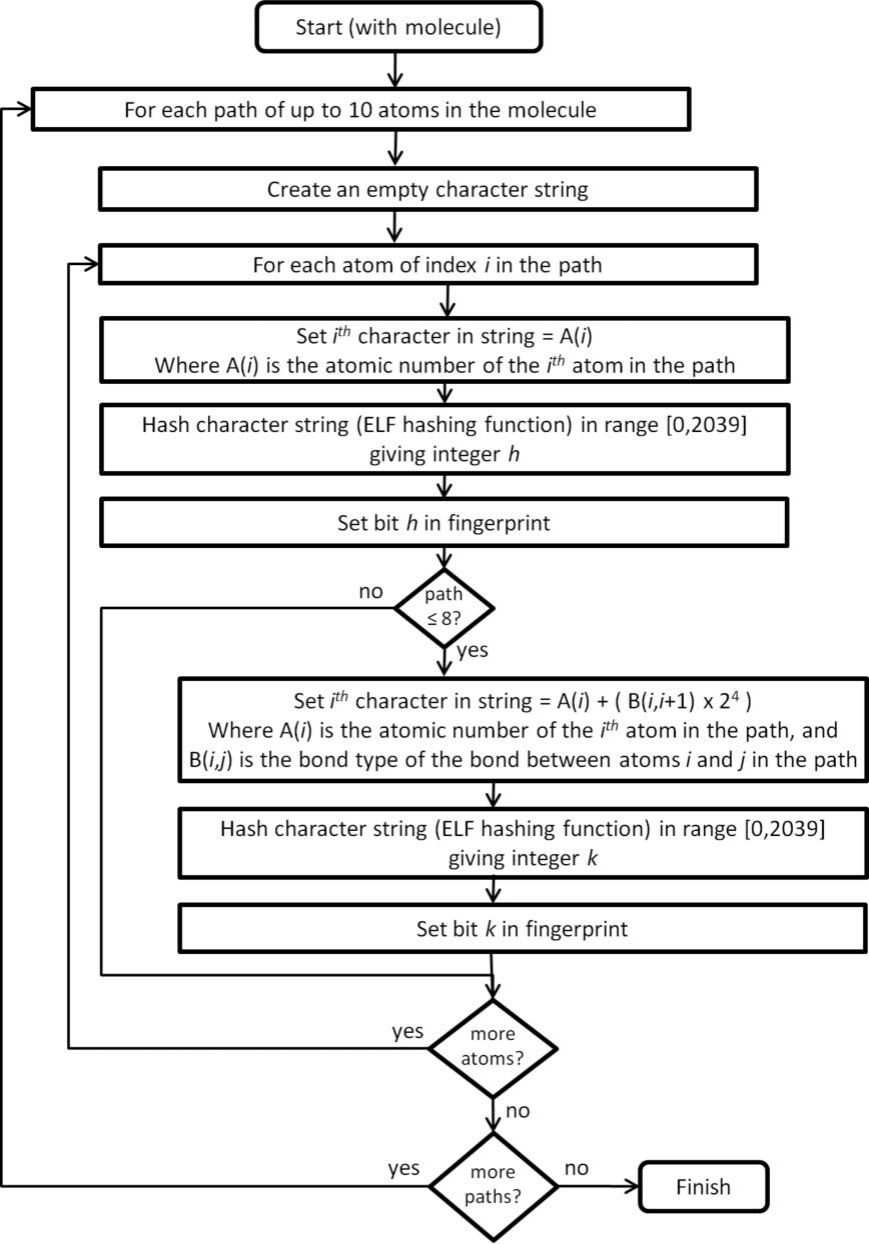
Flow chart explaining the algorithms that create the molecular fingerprints for similarity searching using bonded paths of up to 10 atoms.

The similarity calculation in *WebCSD* uses molecular fingerprints that are determined using the chemical features of the molecules, including atom types, bond types and bonded paths through the molecule. Similarity fingerprints in the CSD are similar to those used in Relibase (Hendlich *et al.*, 2003 ▶). For each separate molecule (or connectivity) in a crystal structure a molecular fingerprint of 2040 bits is generated. The molecular fingerprint is created using all atom and bond paths of up to ten atoms in a molecule. The algorithms used are summarized in Fig. 1 ▶. These algorithms are applied to a given molecule to set bits in the fingerprint. The approach is similar to others used in common chemical search systems [for example, Daylight fingerprints (James & Weininger, 2008 ▶)]. The fingerprints of all the unique connectivities in the CSD are pre-calculated and stored in a relational database so that searching the information is extremely quick.

The quantification of similarity between molecular fingerprints is performed using standard similarity measures. Many articles discuss and compare coefficients for database screening (Whittle *et al.*, 2003 ▶; Haranczyk & Holliday, 2008 ▶). Currently, the Tanimoto (1957 ▶) and Dice (1945 ▶) coefficients are presented to users, both of which produce coefficient values between zero and one (a value of zero indicating no similarity and a value of one indicating identical fingerprint representations). These coefficients have been found to be of the most use for the CSD problem domain, but other measures could be added in the future, such as the Ochiai/cosine similarity (Ochiai, 1957 ▶) or Hamming (1950 ▶) distance.

**Figure 2:**
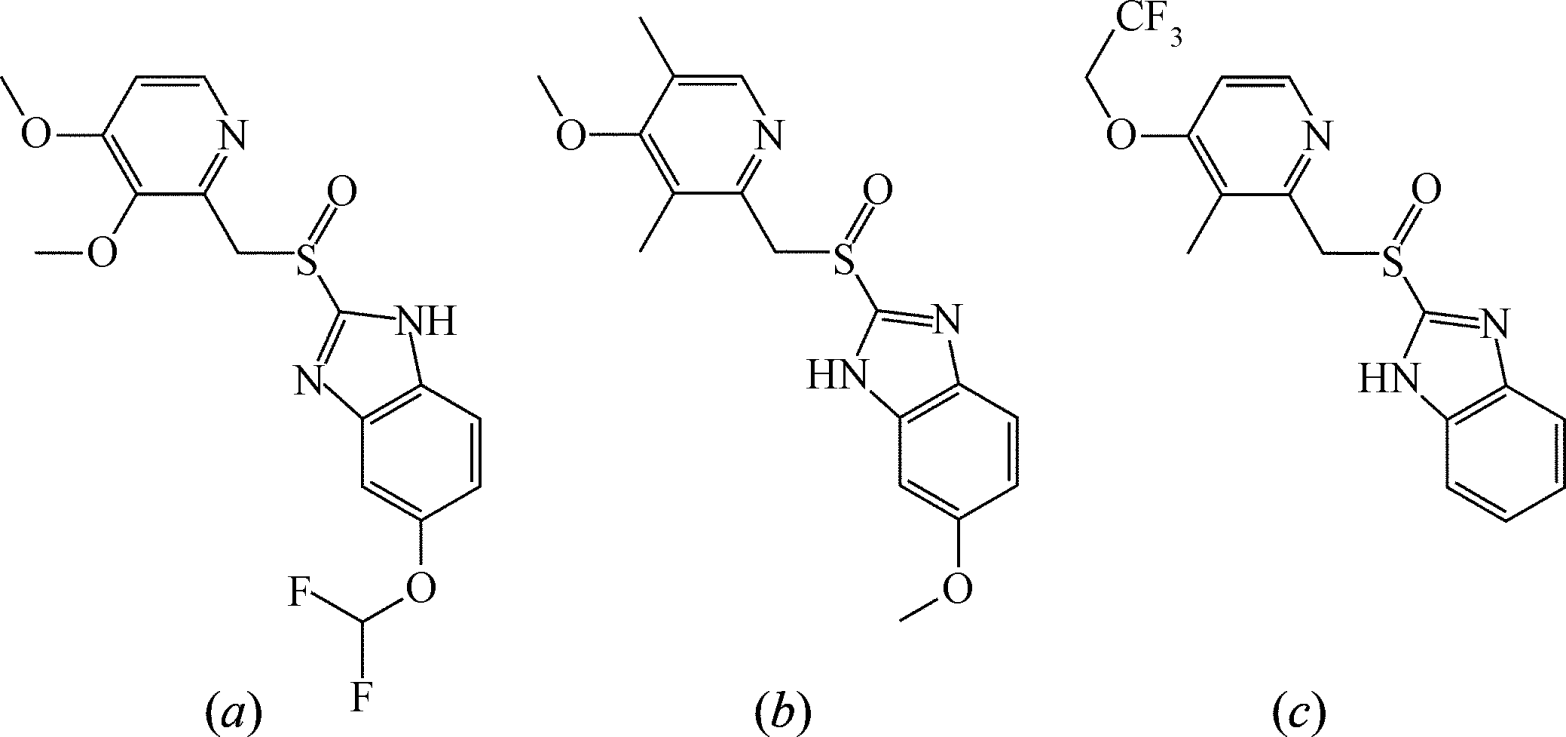
The similarity search results for the drug compound pantoprazole (*a*) include two other drugs with known anti-ulcerative activity: (*b*) omeprazole (CSD refcode UDAVIF; Bhatt & Desiraju, 2007 ▶) with a similarity coefficient of 0.82 and (*c*) lansoprazole (XEGTIM; Vyas *et al.*, 2000 ▶) with a similarity coefficient of 0.70.

The utility of the similarity search tool can be illustrated by taking the top ten selling small-molecule drugs [based on 2006 sales figures in USD (Humphreys, 2007 ▶)] and running a similarity search for each of these. For these ten molecular structures (see supplemental material^1^) a similarity search was performed and the top ten chemically distinct matches, based on the Tanimoto coefficient, were recorded. To determine how relevant the results of these searches were, we can use the ‘bioactivity’ field in the CSD records as a marker for activity (albeit an imperfect one). On average, three out of the ten similarity search results (a total of 30) were listed in the CSD as having bioactivity. The 30 ‘hits’ were manually inspected and 21 of these 30 ‘hits’ had activities obviously related to that of the drug (*e.g.* the similarity search based on Protonix, or pantoprazole, found two other drugs with known anti-ulcerative activity; Fig. 2 ▶).

**Figure 3:**
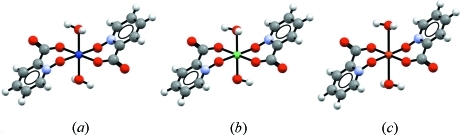
The three-dimensional molecular structures of three diaquabis(picolinato-*N*-oxide) transition metal complexes with the following metal centres: (*a*) Co^2+^ (CSD refcode BIVWOS), (*b*) Ni^2+^ (BIVWUY) and (*c*) Cu^2+^ (BIVXAF).

As with all similarity fingerprints, the fingerprints used for CSD similarity searching have certain strengths and weaknesses. The example above shows that for typical feature-rich drug-like mol­ecules, fingerprints can show reasonable retrieval of related compounds. Because of the nature of the fingerprints, the similarity search will tend to find matches that contain closely related scaffolds. There are, however, a number of caveats with the fingerprints and similarity calculations as currently implemented. First, molecules that contain fewer atoms are less well defined, and as such are more prone to low similarity. Secondly, the fingerprints do not account for cyclicity; for example, hexane and cyclo­hexane are indistinguishable. Thirdly, the fingerprints are element based. Consequently, atoms with related properties, such as halogens, are treated as distinct from one another. This can cause a similarity search to miss what would appear to be chemically reasonable hits. Different transition metal elements are treated as distinct even if they occupy chemically similar environments. For example, consider a search for the molecule shown in Fig. 3 ▶(*b*). One would expect this complex to be highly related to the equivalent Co^2+^ and Cu^2+^ complexes (Figs. 3 ▶ *a* and 3 ▶ *c*; Knuuttila, 1982 ▶), as they have chemically identical scaffolds, but at the moment these would not be listed with high similarity coefficients. In future versions of *WebCSD* we will address such issues by providing alternative generalized fingerprints to address such cases.

### 4.4. Text/numeric searching

One of the benefits of using SQLite is that the text fields in the database can be fragmented (or ‘tokenized’) and stored in a relational database format using the built-in module ‘FTS3’ for full text search indexing. This tokenization and indexing using ‘Google-like’ technology (Hipp, 2006 ▶) means that text searches are extremely fast, as the search engine does not need to read all the text, but simply look up the matching entries in the relevant table. Standard bibliographic information is easily searchable in *WebCSD* including author, journal, publication year, journal volume and page number. The real power of the full text search indexing is more apparent though when using the ‘all text’ search – searches for an exact string such as ‘antibacterial’ (786 hits) or ‘agonist’ (215 hits) take less than a second to search through nearly half a million entries in the CSD.

A number of new specific fields are also available for flexible and/or combined searching, such as bioactivity, habit, phase transitions and polymorphism. The interface is designed such that any of the text/numeric search types can be combined into composite queries, so it is simple to design a search, for example, where the habit field contains the string ‘needle’ and the phase transitions field has any defined value. Further options in the text/numeric interface include a range of advanced date search fields in addition to the year of publication. Users can therefore search based on when structures were added to the CSD or when they were last modified; this means it is simple to re-run an old search, *e.g.* for all bioactive compounds with a plate habit, but restricted to only the entries added since the user last accessed *WebCSD*.

### 4.5. Reduced cell searching

The use of the Niggli reduced cell (generally referred to as ‘the reduced cell’) can be problematic as a result of mathematical instabilities. Whilst the reduced cell can be uniquely defined for any specific lattice, the angles of the reduced cell can vary a great deal with only small changes in the lattice parameters (Andrews *et al.*, 1980 ▶). This problem was avoided in *ConQuest* by using only the reduced cell lengths for searches. The reduced unit cell search algorithm used in *WebCSD* is a new implementation which uses a more advanced methodology involving ‘nearly Buerger reduced cells’ (Andrews & Bernstein, 1988 ▶). Essentially the reduced cell and a set of closely related cells are determined and the database is searched for any matches to this set. The result is that the search tool in *WebCSD* takes full account of the reduced cell angles and thus produces fewer false positive hits when searching the database.

This facility is ideal for experimental crystallographers. The unit cell of a sample is usually determined through analysis of a small data set prior to starting a full experiment. This unit cell can then be compared with entries in the CSD using the reduced cell search in *WebCSD*. This should ensure that organic and metal–organic crystal structures are not redetermined unknowingly.

## 5. Tools for analysing results in *WebCSD*

### 5.1. Embedded three-dimensional viewing

**Figure 4:**
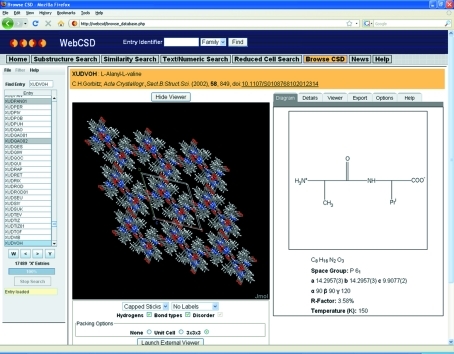
Image of the *WebCSD* results browser showing a 3 × 3 × 3 unit cell packing range for CSD refcode XUDVOH (l-alanyl-l-valine) in the *Jmol* embedded viewer. Channels through the crystal structure formed in the middle of hydrogen-bonded helices can be observed down the crystallographic *c* axis.

The crystal structure information accessed either through searches or simply by browsing the CSD is inherently focused on three-dimensional information – *i.e.* the crystal structure coordinates. This means that it is very important to provide the ability within the results browser for users to access basic three-dimensional molecular and crystal-packing visualization functionality. *WebCSD* allows the use of either of two different three-dimensional viewers as embedded Java applets in the interface: *Jmol* (2009 ▶) or *OpenAstexViewer* (2009 ▶). These molecular viewers both provide a range of atom/bond style options as well as atom labelling and tools to measure distances, angles and torsion angles. *Jmol* also supports some crystallographic options such as the display of a full unit cell or a packing range of 3 × 3 × 3 unit cells. Fig. 4 ▶ shows an example of a 3 × 3 × 3 unit cell packing range for a nanoporous dipeptide crystal structure (CSD refcode XUDVOH; Görbitz, 2002 ▶) where channels can be seen running along the crystallographic *c* axis.

The embedded viewers allow *WebCSD* to be used without the need for additional client-side applications. Users can, however, still choose to export crystal structures from *WebCSD* into *Mercury* (either one structure as a CIF or many as a list of refcodes) for more advanced structure viewing and analysis tools. In this way *WebCSD* can act as a springboard for more complex studies – allowing very fast searches with links to CCDC applications, or exporting of files for other programs, to allow further investigation of the results.

### 5.2. Hyperlinking of textual results

**Figure 5:**
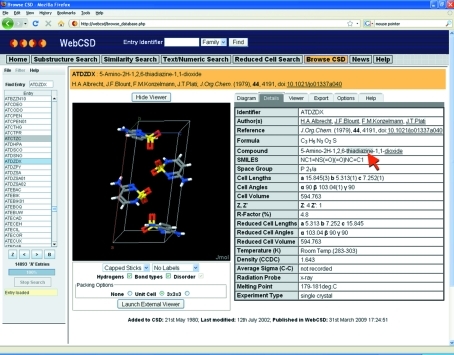
Hyperlinking within the results browser for CSD entry ATDZDX. The red arrow indicates the ‘thiadiazine’ section of the compound name which could be used as the basis of a further text/numeric search.

The results browser in *WebCSD* provides, alongside the three-dimensional viewer, a two-dimensional molecular diagram and a display of the textual and numeric information. Within the ‘Details’ tab of the results browser, bibliographic as well as chemical, crystallographic and experimental information relating to the displayed structure is given. A range of the categories within the ‘Details’ tab are parsed by *WebCSD* to identify useful pieces of text from which to hyperlink. For example, all authors listed for an entry are hyperlinked, such that clicking on their name will launch an author name search. Similarly, compound name and synonym sections are also hyperlinked so that when looking at the structure of 5-amino-2H-1,2,6-thiadiazine-1,1-dioxide (CSD refcode ATDZDX; Albrecht *et al.*, 1979 ▶), for example (Fig. 5 ▶), it is possible to simply click on ‘thiadiazine’ and, in less than a second, find all 154 structures in the CSD with the string ‘thiadiazine’ in their compound name. This facility makes browsing between entries with similar chemistries particularly efficient. When present, the DOI (data object identifier) is hyperlinked, allowing access to the original publication of the crystal structure.

## 6. Documentation, availability and environment

*WebCSD* includes a set of FAQs and pop-up help messages within the interface. Access to a 500-structure subset of the CSD using *WebCSD* is freely available from the CCDC website (http://www.ccdc.cam.ac.uk/free_services/teaching/) for demonstration or teaching purposes. *WebCSD* is currently fully supported on the following web browsers: Internet Explorer (version 7.0 and later), Mozilla Firefox (version 2.0 and later) and Safari (version 4.0 and later). The software has also been tested and shown to work on a number of other common browsers including Google Chrome. *WebCSD* access is provided *via* a public server hosted at the CCDC to CSD system subscribers holding unlimited seat licences. For more information about access, contact admin@ccdc.cam.ac.uk.

## Supplementary Material

Zip file containing the molecular structures of the top ten selling small-molecule drugs in SDF format. DOI: 10.1107/S0021889810000452/kk5057sup1.zip

## Footnotes

1 Supplementary data for this paper are available from the IUCr electronic archives (Reference: KK5057). Services for accessing these data are described at the back of the journal.
